# Clinical and Genetic Characterization of Esophageal Atresia: A Contemporary Cohort Integrating Phenotyping and Genomic Testing

**DOI:** 10.3390/genes17060654

**Published:** 2026-06-01

**Authors:** Purificacion Marin-Reina, Irene Reig Talamante, Anna Parra Llorca, Inmaculada Navarro Escandell, Carla Martin Grau, Angel Zuñiga Cabrera, Cinta Navarro Moreno, Alba Gabaldon Albero, Carmen Orellana Alonso, Monica Rosello Piera, Pilar Saenz Gonzalez, Francisco Martinez Castellano

**Affiliations:** 1 Dysmorphology and Pediatric Rare Diseases, Division of Neonatology, Hospital Universitari i Politècnic La Fe (HULAFE), 46026 Valencia, Spain; marin_pur@gva.es; 2 Neonatal Research Group, Instituto de Investigación Sanitaria La Fe (IISLAFE), 46026 Valencia, Spain; reig_iretal@gva.es (I.R.T.); parra_ann@gva.es (A.P.L.); saenz_pilgon@gva.es (P.S.G.); 3 Division of Neonatology, Hospital Universitari i Politècnic La Fe (HULAFE), 46026 Valencia, Spain; 4 Facultad de Medicina, Universitat de València, 46010 Valencia, Spain; inaes2@alumni.uv.es; 5 Genetics Unit, Hospital Universitari i Politècnic La Fe (HULAFE), 46026 Valencia, Spain; martin_carlagra@gva.es (C.M.G.); zunyiga_ang@gva.es (A.Z.C.); orellana_car@gva.es (C.O.A.); rosello_mpi@gva.es (M.R.P.); 6 Translational Genetics Research Group, Instituto de Investigación Sanitaria La Fe (IIS La Fe), 46026 Valencia, Spain; alba_gabaldon@iislafe.es; 7 Biomedical Network Research Center for Rare Diseases (CIBERER), Instituto de Salud Carlos III (ISCIII), 28029 Madrid, Spain; cinta_navarro@iislafe.es; 8 Molecular, Cellular and Genomics Biomedicine Research Group, Instituto de Investigación Sanitaria La Fe (IIS La Fe), 46026 Valencia, Spain

**Keywords:** esophageal atresia, tracheoesophageal fistula, prenatal diagnosis, genetic testing, phenotype-guided approach, VACTERL association, neurodevelopment, mortality

## Abstract

**Background:** Esophageal atresia (EA) is a complex congenital anomaly frequently associated with additional malformations and genetic conditions. Despite advances in prenatal imaging and genomic technologies, establishing an etiologic diagnosis and performing accurate risk stratification remain challenging due to marked clinical and genetic heterogeneity. **Methods:** We conducted a retrospective cohort study of neonates diagnosed with EA and admitted to a level IIIc neonatal intensive care unit between 2005 and 2024. Prenatal findings, associated anomalies, genetic testing results, mortality, and neurodevelopmental outcomes beyond 12 months were analyzed. **Results:** A total of 105 neonates were included, of whom 10.5% were diagnosed prenatally. Isolated EA was identified in 55.2% of patients, whereas 44.8% had associated anomalies, most commonly congenital cardiac defects. Clinically relevant genetic findings were identified in 10.5% of the total cohort (23.4% of complex EA cases). These findings reflect a clinically selected subgroup and should not be interpreted as diagnostic yields applicable to unselected populations or as a comparison between testing modalities. Overall mortality was 11.4%. Lower birth weight showed the strongest association with mortality in univariable analyses; however, no independent predictors were inferred due to the limited number of events. All deceased patients had complex malformative conditions and/or extreme prematurity. Among children with follow-up beyond 12 months, 88.5% demonstrated age-appropriate neurodevelopment. **Conclusions:** EA is characterized by substantial etiologic and phenotypic heterogeneity. Prenatal detection remains challenging, although advances in fetal imaging may improve diagnostic accuracy. A phenotype-guided approach integrating clinical evaluation and genetic testing may support etiologic diagnosis, recurrence counseling, and follow-up planning in selected patients. However, because testing was indication-driven and evolved over time, the reported diagnostic yields should not be generalized to unselected EA populations or interpreted as comparative performance across testing modalities.

## 1. Introduction

Esophageal atresia (EA) is one of the most common major congenital malformations, with an estimated incidence in Europe of 2.43 per 10,000 live births [1,2]. EA is defined as a congenital interruption of esophageal continuity, with or without communication with the trachea. Approximately 86% of cases present with distal tracheoesophageal fistula, 7% lack tracheoesophageal communication, 2% present with proximal fistula, and 4% correspond to isolated tracheoesophageal fistula without atresia [3].

Prenatal diagnosis remains challenging, particularly in cases with distal tracheoesophageal fistula, which account for the majority of cases. In this subgroup, amniotic fluid volume is frequently normal due to the passage of fluid through the fistula into the stomach, which limits the sensitivity of indirect ultrasound findings. Consequently, classical signs such as polyhydramnios or an absent gastric bubble are inconsistent and may be absent, especially in isolated cases. Detection rates vary widely between centers, ranging from 10% to 50% [4]. Advances in perinatal management and surgical care have improved survival rates, which now approach 90% [5]. Mortality risk is strongly associated with low birth weight and major congenital heart disease [6,7]. These factors, together with genetic abnormalities, also influence long-term neurodevelopmental outcome [8].

Approximately 50% of patients have associated anomalies [4,5], most commonly cardiovascular defects and features included in the VACTERL (Vertebral, Anorectal, Cardiac, Tracheoesophageal, Renal, Limb anomalies) association [6]. EA demonstrates marked etiologic heterogeneity, including chromosomal abnormalities, copy-number variants, and monogenic syndromes. Furthermore, genetic alterations are identified in 6–10% of cases, depending on patient selection and testing strategies [7,8]. Consequently, the role of broad genomic testing in unselected patients is unclear. There is a need for strategies that integrate phenotypic assessment with targeted genetic testing and are guided by clinical factors.

The recurrence risk increases by 0.5–2% in families with one affected child and up to 20% in families with more than one affected child [2].

In this context, the present study aimed to provide a comprehensive clinical and genetic characterization of a contemporary cohort of patients with EA, with particular emphasis on the relationship between phenotypic presentation, diagnostic yield of genetic testing, prenatal findings, and neurodevelopmental outcomes.

**Figure 1:**
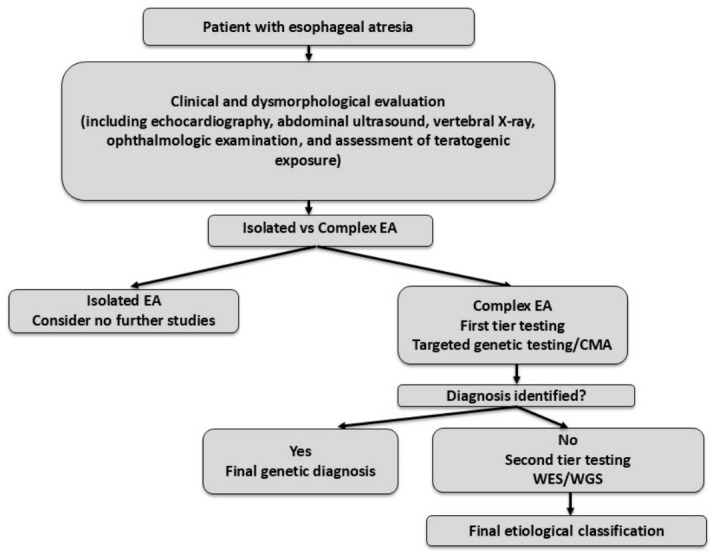
Expert-informed conceptual framework for genetic evaluation in esophageal atresia based on retrospective findings and institutional clinical practice. Clinical evaluation distinguishes isolated from complex EA and guides stepwise genetic testing, including karyotype, chromosomal microarray analysis (CMA), targeted testing, and exome/genome sequencing when no diagnosis is established. Abbreviations: EA, esophageal atresia; CMA, chromosomal microarray analysis; WES, whole-exome sequencing; WGS, whole-genome sequencing.

Based on these considerations, we present an expert-informed framework for phenotype-guided genetic evaluation, integrating clinical assessment and genetic testing strategies (Figure 1).

## 2. Materials and Methods

We conducted a retrospective cohort study including all neonates diagnosed with esophageal atresia (EA) (ICD-10 codes Q39.0 and Q39.1) admitted to a level IIIc neonatal intensive care unit between 2005 and 2024. Data were collected from hospital electronic medical records and primary care follow-up reports.

The variables analyzed included prenatal suspicion of EA, gestational age (defined as completed weeks of gestation at delivery), birth anthropometric measurements, associated malformations, genetic testing, mortality, and neurodevelopmental outcomes assessed at the last available follow-up visit after a minimum follow-up period of 12 months. Maternal and prenatal risk factors were not systematically included due to incomplete or inconsistent documentation.

Cases were classified as isolated EA when no additional anomalies were identified and as complex EA when at least one associated malformation was present. VACTERL association was defined by the presence of at least three component anomalies in the absence of an alternative diagnosis.

Mortality was analyzed as a binary outcome (deceased vs. survivors). Given the limited number of mortality events (n = 12) and the clinical heterogeneity of affected patients, multivariable regression modeling was considered statistically unreliable and was not performed. Comparative analyses were therefore restricted to univariable analyses using Student’s *t*-test or Mann–Whitney U test for continuous variables and chi-square or Fisher’s exact test for categorical variables, as appropriate.

Genetic testing was requested in accordance with institutional protocols and guided by clinical findings. Testing strategies were indication-driven and evolved over time in line with advances in genomic technologies. However, this approach introduces selection bias and precludes direct comparison of the diagnostic yield of different testing methods. Variant interpretation followed the American College of Medical Genetics and Genomics/Association for Molecular Pathology (ACMG/AMP) guidelines, and variants of uncertain significance were not considered diagnostic.

Statistical analysis was performed using SPSS software (version 20.0; IBM Corporation, Armonk, NY, USA). Continuous variables were expressed as the mean ± standard deviation or the median (interquartile range), depending on the distribution, and were compared using a Student’s *t*-test or a Mann–Whitney U-test. Categorical variables were compared using the chi-square test or Fisher’s exact test, as appropriate. All tests were two-sided, and a *p*-value < 0.05 was considered statistically significant.

Missing data were minimal; follow-up information was unavailable for two patients, who were excluded from the neurodevelopmental analysis. The relatively small size of some subgroups may limit statistical power, and the results should be interpreted with caution.

## 3. Results

### 3.1. Prenatal Findings and Perinatal Characteristics

During the study period, 105 neonates with EA were included, 61.9% of whom were male. The demographic and prenatal characteristics of the study population are summarized in Table 1.

**Figure 2:**
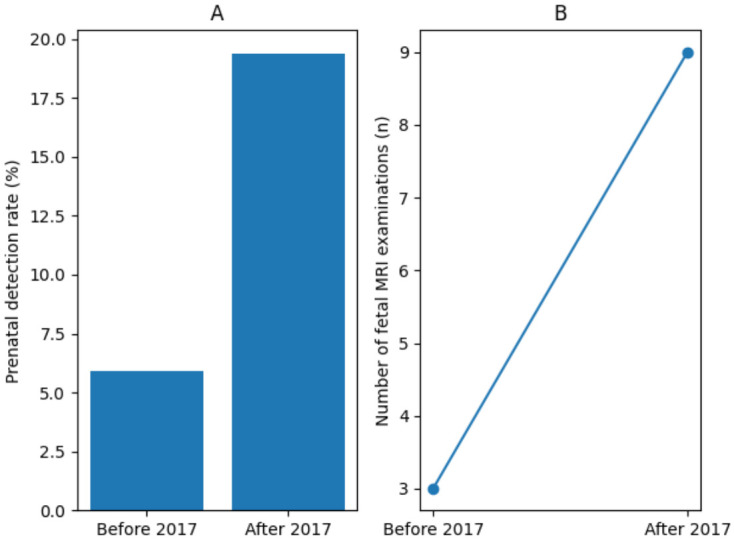
Prenatal detection and MRI use in EA. Prenatal detection of esophageal atresia and use of fetal magnetic resonance imaging (MRI) before and after 2017. (**A**) Prenatal detection rate (%) increased from 5.9% before 2017 to 19.4% after 2017 (*p* = 0.071). (**B**) The number of fetal MRI examinations increased from 3 to 9 cases and was associated with confirmed prenatal diagnosis (*p* < 0.001).

Abnormalities on prenatal ultrasound were detected in 50 pregnancies (47.5%). Prenatal diagnosis of EA was established in 11 patients (10.5%). The detection rate increased from 5.9% in births before 2017 to 19.4% thereafter, although this trend did not reach statistical significance (*p* = 0.071), likely due to limited sample size. The introduction of fetal MRI was strongly associated with confirmed prenatal diagnosis (*p* < 0.001) (Figure 2).

### 3.2. EA Type and Associated Anomalies

Regarding EA subtype, 94 cases (89.5%) had distal fistula, 9 (8.6%) had no tracheoesophageal communication, and 2 (1.9%) presented with proximal fistula.

Isolated EA was identified in 58 patients (55.2%), whereas 47 (44.8%) had complex EA. A total of 91 associated anomalies were identified. Cardiac defects were the most frequent (27.5%), followed by genitourinary (19.8%) and digestive (13.2%), while vertebral (8.8%), limb (8.8%), and craniofacial (8.8%) anomalies were also common. Other detected anomalies were cerebral (5.5%), ocular (4.4%) and auditory (3.3%). According to the malformative pattern, patients were classified into the following groups: isolated EA (55.2%), EA with a single additional VACTERL component (14.2%), VACTERL association (12.4%), and other congenital anomaly conditions (18.1%).

### 3.3. Genetic Testing and Etiological Diagnosis

Genetic testing was performed in 38 of 47 patients with complex esophageal atresia (80.9%) based on clinical indication. Testing strategies were non-uniform and indication-driven, evolving over time. This reflects the fact that the cohort was clinically selected, which precludes direct comparison of the diagnostic yield of different modalities.

Apparent differences in diagnostic yield across testing modalities were observed, with higher yields in targeted molecular testing (80.0%) and more modest yields in broader genomic approaches such as clinical exome (28.6%) and whole-exome sequencing (25.0%). However, these findings should be interpreted with substantial caution. Testing was performed in a non-systematic manner based on clinical suspicion, and diagnostic strategies evolved considerably during the study period. Consequently, patients undergoing targeted testing often had stronger syndromic suspicion, whereas broader genomic approaches were typically used in more heterogeneous unresolved cases. Therefore, these observed yields largely reflect selection bias and phenotypic enrichment rather than the intrinsic diagnostic performance of specific testing modalities.

A diagnostic genetic alteration was identified in 11 patients (10.5% of the total cohort), corresponding to 23.4% of patients with complex EA. Parental segregation analysis of variants in autosomal dominant genes confirmed de novo status in all cases except one familial case of Feingold syndrome due to an inherited *MYCN* variant (Table 2).

One patient carried a likely pathogenic variant in *HNRNPH1* that was consistent with the observed phenotype. Two incidental findings were identified (a recurrent 16p13.11 microduplication and a balanced translocation, *t*(8;13)). Additionally, two patients carried variants of uncertain significance in *NKX2-5* (NM_004387.4:c.839C>T; p.Pro280Leu) and *MAMLD1* (c.2170C>G; p.Leu724Val).

A hierarchical approach integrating clinical and genetic data was used to classify patients into mutually exclusive etiological categories. A confirmed genetic diagnosis was established in 11 patients (10.5%), while 17 patients (16.2%) had a recognizable clinical association without molecular confirmation. Nineteen patients (18.1%) showed a non-specific malformative pattern, and 58 patients (55.2%) were *classified* as isolated EA (Table 3).

Among patients with isolated EA, 25 underwent genetic testing; however, no pathogenic or likely pathogenic variants were identified. This finding reinforces the very low diagnostic yield of genetic testing in non-syndromic isolated EA.

A familial case involving an affected father and son was identified, but whole-exome sequencing did not reveal any pathogenic variants.

Genetic diagnoses contributed to etiological classification, informed recurrence risk counseling, and may support individualized follow-up, particularly in syndromic cases.

### 3.4. Mortality and Risk Factors

Twelve patients died (11.4%). Notably, all the deceased patients had complex EA (100%). The median age at death was 17 days (interquartile range: 1.8–88 days), and one case was type I esophageal atresia. All deceased patients had additional comorbidities considered clinically relevant to their unfavorable course (Table 4). Three deaths were attributed to postoperative complications (pneumothorax with mediastinitis, haemodynamic instability, and bronchial fistula).

A comparative analysis between deceased and surviving patients is summarized in Table 5. Deceased patients had significantly lower gestational age (34.2 ± 4.1 vs. 37.4 ± 2.6 weeks, *p* = 0.005) and lower birth weight (1740 ± 577 vs. 2580 ± 666 g, *p* < 0.001) compared with survivors. Major cardiac anomalies were more frequently among deceased patients; however, a statistically significant association with mortality could not be established, likely due to small sample size and the heterogeneity in cardiac defect classification. The proportion of multiple gestations did not differ significantly between groups (16.7% vs. 9.8%, *p* = 0.61).

Mortality occurred almost exclusively in patients with associated malformations and complex clinical conditions. All deceased patients had clinically relevant comorbidities contributing to their unfavorable outcomes. Given the limited number of events, these findings should be interpreted descriptively.

### 3.5. Neurodevelopmental Outcomes

Neurodevelopmental outcomes were assessed during routine follow-up by primary care pediatricians and at specialized dysmorphology clinics using the Haizea–Llevant developmental scale. Patients with suspected developmental delay or clinical concern were evaluated further by pediatric neurologists. Formal standardized neurodevelopmental testing was not systematically applied, which may have led to underestimation of subtle impairments. This approach may preferentially detect moderate-to-severe impairment while underdetecting subtle neurocognitive deficits.

Of the 93 surviving patients, two were excluded from the follow-up analysis due to transfer abroad before 1 year of age. Among the remaining 91 patients with follow-up beyond 12 months, neurodevelopmental impairment was identified in 11 children, predominantly among those with complex EA, while most children (88.5%) demonstrated age-appropriate neurodevelopment. Given the absence of systematic standardized assessment, subtle deficits may have been underrecognized, and these results should be interpreted with caution.

## 4. Discussion

In this contemporary cohort, EA emerges as a clinically heterogeneous condition in which genetic diagnosis is largely driven by phenotype rather than testing modality.

Prenatal diagnosis of esophageal atresia remains challenging, particularly in cases with distal tracheoesophageal fistula. While the overall detection rate in our cohort was low (10.5%), we observed an increase over time, likely reflecting advances in prenatal imaging and improved recognition of indirect sonographic signs. The association between fetal MRI and confirmed diagnosis in our series highlights its role as a complementary tool following ultrasound suspicion, particularly in complex or syndromic cases. Previous studies have reported intrauterine diagnosis rates of up to 31.7%, with markedly higher detection rates in cases of EA without fistula (77.9%) than in cases with distal fistula (21.9%) [9]. However, reported detection rates vary widely across centers, ranging from 10% to 50% [10]. Our findings are consistent with prior studies showing that fetal MRI improves diagnostic accuracy and is usually employed to confirm findings after an initial ultrasound scan raises concerns [11,12].

Late prematurity and low birth weight were common, consistent with previous studies [7,8]. Multiple gestations accounted for 10.5% of cases, supporting an increased relative risk of congenital anomalies among twin pregnancies [13,14].

In our cohort, isolated EA accounted for 55.2%, similar to that reported in other series [4,5]. Associated anomalies were common, especially cardiovascular defects, followed by skeletal, gastrointestinal, and renal anomalies, as reported in other series [6,8]. The predominance of cardiac anomalies reinforces the need for systematic cardiologic evaluation.

VACTERL association was identified in 27.7% of patients with complex EA, within the range reported in the literature, although the data are variable, from 51–53% [4,8] to 23% [5], likely reflecting variability in diagnostic criteria [15].

Genetic testing plays an increasingly important role in esophageal atresia (EA) [9], although diagnostic yields should be interpreted cautiously. In our cohort, observed yields reflected a clinically selected population and indication-driven testing strategies rather than the intrinsic performance of specific genomic techniques. A molecular diagnosis was established in more than one-third of tested patients with complex EA, but these findings should not be extrapolated to unselected populations or interpreted as evidence of superiority between testing modalities. Overall, our results support a clinically stratified approach to genetic evaluation.

Patients with a confirmed genetic diagnosis tended to present more complex phenotypes, reinforcing the value of detailed clinical evaluation. Consistent with previous reports, the most frequent diagnoses in our cohort included aneuploidies such as trisomy 18 and trisomy 21 [16,17], as well as monogenic and microdeletion syndromes [5,18]. Notably, chromosomal microarray analysis (CMA) did not yield diagnostic findings in our series, likely reflecting historical testing strategies, patient selection, and the use of alternative techniques such as MLPA during earlier periods rather than a lack of diagnostic utility. In isolated EA, the diagnostic yield of karyotyping, CMA, and even whole-exome sequencing remains very low [8,13], supporting a likely multifactorial or polygenic basis, although candidate genes such as *PCDH1* and *RAB3GAP2* have been proposed without consistent validation [14]. In contrast, genetic evaluation appears most informative in complex EA, where karyotyping may confirm clinically suspected aneuploidies [2], CMA can identify copy-number variants in a minority of cases [1,2], and targeted or broader genomic approaches may be useful when syndromic suspicion persists despite negative first-tier testing. These estimates should be interpreted cautiously given variability in patient selection and study design.

When first-tier testing is inconclusive and clinical suspicion remains, adopting broader genomic approaches such as WES or whole-genome sequencing can increase the diagnostic yield, up to 26% in selected cohorts [2,19]. However, compared with other congenital malformations, the diagnostic yield of WES in EA remains lower than that reported for other congenital malformations, suggesting that additional genetic and non-genetic mechanisms remain incompletely understood [20]. In patients meeting criteria for the VACTERL association, the overall diagnostic yield of genetic testing ranges from 0% to 16% [2,19,21], and most cases (approximately 90%) are sporadic [21].

Historically, mortality in EA has been associated with low birth weight, prematurity, and major cardiac anomalies. The prognostic importance of these factors was first described by Waterson et al. in 1962 [22], and subsequent refinements, including more recent classifications [23], continue to identify very low birth weight and complex congenital heart disease as principal determinants of survival. In our cohort, mortality was concentrated among patients with prematurity, lower birth weight, and major associated comorbidities, including complex congenital anomalies, which is consistent with previous literature. Lower birth weight showed the strongest association with mortality in comparative analyses. However, given the limited number of deaths and the marked clinical heterogeneity of affected patients, multivariable modeling was considered statistically unreliable and was not used to infer independent predictors. Therefore, these findings should be interpreted descriptively and as hypothesis-generating rather than confirmatory.

The overall survival rate (85.9%) was slightly lower than that reported in some contemporary series [24], possibly reflecting methodological differences, such as including patients who died before undergoing surgery.

Neurodevelopmental outcomes were generally favorable [25], with impairment mainly observed in syndromic or polymalformative cases. This highlights the importance of long-term multidisciplinary follow-up. However, the absence of systematic standardized assessment likely led to the underestimation of subtle impairments; therefore, these findings should be interpreted cautiously as a conservative estimate of neurodevelopmental outcome. Further studies incorporating standardized neurodevelopmental assessments are required to provide a clearer picture of long-term outcomes in this population.

Our study provides a contemporary clinical and genetic characterization of EA in routine practice. Figure 1 illustrates an expert-informed conceptual framework for genetic evaluation in clinically selected patients with EA.

This study has several limitations. The retrospective design and long study period resulted in variability in clinical management and genetic testing strategies. Heterogeneity in genetic testing strategies represents one of the main limitations of this study. Testing was not systematically applied across the cohort, indications were determined by clinical suspicion, and testing methodologies changed substantially over the study period. These factors introduced significant selection bias and methodological heterogeneity, limiting direct comparisons between testing modalities and preventing generalization of observed diagnostic yields to broader EA populations. Additionally, the proposed diagnostic workflow was not prospectively validated and should be interpreted as a conceptual framework rather than a data-derived clinical algorithm.

Overall, our findings highlight the value of integrating detailed phenotypic assessment with genetic testing in clinically selected patients, while emphasizing the need for prospective studies using standardized genomic approaches.

## 5. Conclusions

EA is a complex congenital condition that requires multidisciplinary management. Although prenatal diagnosis remains challenging, advances in fetal imaging and the complementary use of magnetic resonance imaging may improve diagnostic accuracy in selected cases.

Once EA is suspected, a systematic evaluation is essential to identify associated malformations—particularly those within the VACTERL spectrum. Prognosis appears to be strongly influenced by birth weight, major cardiac anomalies, and the presence of genetic or syndromic conditions.

Early, phenotype-guided genetic evaluation and comprehensive assessment of associated anomalies may support etiologic diagnosis and recurrence counseling in selected patients with esophageal atresia. However, optimal diagnostic pathways require prospective multicenter validation using standardized genetic testing strategies.

## Figures and Tables

**Table 1:** Prenatal and perinatal characteristics of patients with esophageal atresia (n = 105).

Variable	Value
**Demographic characteristics**	
Total patients	105
Male sex	65 (61.9%)
Multiple pregnancy	11 (10.5%)
**Perinatal characteristics**	
Gestational age, weeks	37.0 ± 2.9
Birth weight, g	2484 ± 706
Small for gestational age	16 (15.2%)
**Prenatal findings**	
Polyhydramnios	50 (47.5%)
Prenatal diagnosis of EA	11 (10.5%)
Fetal MRI performed	12 (11.4%)

Values are presented as number (%) or mean ± standard deviation.

**Table 2:** Genetically confirmed diagnoses in patients with esophageal atresia.

Diagnosis	N	Gene	Variant(s) (HGVS Nomenclature)	Classification	Diagnostic Test
Trisomy 18	1	NA	47,XX,+18	Pathogenic	Karyotype
Trisomy 21	1	NA	47,XX,+21	Pathogenic	Karyotype
22q11 deletion syndrome	1	NA	22q11.2 deletion	Pathogenic	MLPA
CHARGE syndrome	3	*CHD7*	c.5534+1G>T	Pathogenic	Sanger sequencing
			c.343dup (p.His115Profs*18)	Likely pathogenic	Clinical exome
			c.4644+1G>A	Pathogenic	Whole-genome sequencing
Feingold syndrome	2	*MYCN*	c.1171C>T (p.Arg391Cys)	Pathogenic	Sanger sequencing
			c.1147C>T (p.Arg383Cys)	Likely pathogenic	
AEG syndrome	1	*SOX2*	Intragenic deletion	Pathogenic	Semi-quantitative PCR
Fanconi anemia	1	*FANCA*	Compound heterozygous: c.[3788_3790delTCT; c.2303T>C] (p.[Phe1263del; p.Leu768Pro])	Pathogenic/ Likely pathogenic	Clinical exome
Neurodevelopmental disorder (OMIM 620083)	1	*HNRNPH1*	c.880C>T (p.Arg294Trp)	Likely pathogenic	Clinical exome
**Total**	**11**	—	—	—	—

Variants are described according to Human Genome Variation Society (HGVS) nomenclature. Variants of uncertain significance were not considered diagnostic and are reported separately in the text. NA: not applicable. Abbreviations: MLPA, multiplex ligation-dependent probe amplification; PCR, polymerase chain reaction; OMIM, Online Mendelian Inheritance in Man.

**Table 3:** Hierarchical etiologic classification of patients with esophageal atresia.

Diagnostic Category	n (%)
Isolated esophageal atresia	58 (55.2)
Syndromic/genetic diagnosis (confirmed)	11 (10.5)
Recognizable clinical association (non-genetic diagnosis)	17 (16.2)
Non-specific malformative pattern	19 (18.1)
**Total**	**105 (100)**

Values are presented as number (%). Etiological classification was established according to clinical assessment, associated anomalies, and genetic results. Categories are mutually exclusive and were assigned based on a hierarchical classification integrating clinical assessment and genetic findings.

**Table 4:** Individual clinical characteristics of deceased patients with EA.

Case	Birth Weight (g)	Gestational Age (Weeks)	Postnatal Age at Death (Days)	Comorbidities	Genetic Diagnosis
1	600	25	7	Extreme prematurity, anal atresia	None
2	800	27	1	Extreme prematurity	None
3	1495	34	60	Major congenital heart disease	None
4	1620	35	87	Major congenital heart disease	None
5	1680	36	5	Major congenital heart disease	Trisomy 18
6	1800	34	3	Potter sequence	None
7	1860	36	4	Major congenital heart disease	None
8	1960	38	3	Major congenital heart disease	None
9	2000	35	34	Giant omphalocele, ileal atresia, annular pancreas	None
10	2210	38	330	Chronic lung disease, dysphagia, cellular immunodeficiency	22q11.2 deletion
11	2210	35	240	Hydrocephalus, dysphagia	None
12	2650	37	41	Major congenital heart disease	*CHD7* pathogenic variant

Values are presented as individual patient data. All deceased patients had at least one major associated comorbidity considered clinically relevant to their clinical course.

**Table 5:** Comparison between surviving and deceased patients with EA.

Variable	Total (n = 105)	Survivors (n = 93)	Deceased (n = 12)	*p*-Value
Gestational age, weeks	37.0 ± 2.9	37.4 ± 2.6	34.2 ± 4.1	0.005
Birth weight, g	2484 ± 706	2580 ± 666	1740 ± 577	<0.001
Complex EA, n (%)	47 (44.8)	35 (37.6)	12 (100)	<0.001
Multiple gestation, n (%)	11 (10.5)	9 (9.8)	2 (16.7)	0.61

Values are presented as mean ± standard deviation or number (%). Comparisons were performed using Student’s *t*-test or Mann–Whitney *U* test for continuous variables and chi-square or Fisher’s exact test for categorical variables, as appropriate.

## Data Availability

The data presented in this study are available on reasonable request from the corresponding author. The data are not publicly available due to privacy and ethical restrictions.

## Abbreviations

The following abbreviations are used in this manuscript:

EA	Esophageal atresia
TEF	Tracheoesophageal fistula
VACTERL	Vertebral defects, Anal atresia, Cardiac defects, Tracheoesophageal fistula, Renal anomalies, Limb abnormalities
MRI	Magnetic resonance imaging
NICU	Neonatal intensive care unit
SGA	Small for gestational age
CMA	Chromosomal microarray analysis
MLPA	Multiplex ligation-dependent probe amplification
NGS	Next-generation sequencing
WES	Whole-exome sequencing
WGS	Whole-genome sequencing
PCR	Polymerase chain reaction
CNS	Central nervous system
OMIM	Online Mendelian Inheritance in Man
